# Who is in the emergency room matters when we talk about door-to-needle time: a single-center experience

**DOI:** 10.1055/s-0043-1768672

**Published:** 2023-07-04

**Authors:** Alejandro M. Brunser, Juan-Cristobal Nuñez, Eloy Mansilla, Gabriel Cavada, Verónica Olavarría, Paula Muñoz Venturelli, Pablo M. Lavados

**Affiliations:** 1 Clínica Alemana Universidad del Desarrollo, Facultad de Medicina, Departamento de Neurología y Psiquiatría Santiago, Servicio de Neurología, Unidad de Neurología, Santiago, Chile.; 2 Clínica Alemana Universidad del Desarrollo, Facultad de Medicina, Departamento de Emergencias Generales, Santiago, Chile.; 3 Clínica Alemana Universidad del Desarrollo, Departamento de Desarrollo Académico e Investigación, Unidad de Investigación y Ensayos Clínicos, Santiago, Chile.; 4 Clínica Alemana Universidad del Desarrollo, Facultad de Medicina, Departamento de Cuidados Críticos, Santiago, Chile.; 5 Clínica Alemana Universidad del Desarrollo, Facultad de Medicina, Instituto de Ciencias e Innovación en Medicina, Centro de Estudios Clínicos, Santiago, Chile.; 6 University of New South Wales, Faculty of Medicine, The George Institute for Global Health, Sydney, Australia.

**Keywords:** Stroke, Fibrin Clot Lysis Time, Thrombolytic Therapy, Accidente Cerebrovascular, Tiempo de Lisis del Coágulo de Fibrina, Terapia Trombolítica

## Abstract

**Background** The efficacy of intravenous thrombolysis (IVT) is time-dependent.

**Objective** To compare the door-to-needle (DTN) time of stroke neurologists (SNs) *versus* non-stroke neurologists (NSNs) and emergency room physicians (EPs). Additionally, we aimed to determine elements associated with DTN ≤ 20 minutes.

**Methods** Prospective study of patients with IVT treated at Clínica Alemana between June 2016 and September 2021.

**Results** A total of 301 patients underwent treatment for IVT. The mean DTN time was 43.3 ± 23.6 minutes. One hundred seventy-three (57.4%) patients were evaluated by SNs, 122 (40.5%) by NSNs, and 6 (2.1%) by EPs. The mean DTN times were 40.8 ± 23, 46 ± 24.7, and 58 ± 22.5 minutes, respectively. Door-to-needle time ≤ 20 minutes occurred more frequently when patients were treated by SNs compared to NSNs and EPs: 15%, 4%, and 0%, respectively (odds ratio [OR]: 4.3, 95% confidence interval [95%CI]: 1.66–11.5, *p* = 0.004). In univariate analysis DTN time ≤ 20 minutes was associated with treatment by a SN ( *p* = 0.002), coronavirus disease 2019 pandemic period ( *p* = 0.21), time to emergency room (ER) ( *p* = 0.21), presence of diabetes ( *p* = 0.142), hypercholesterolemia ( *p* = 0.007), atrial fibrillation ( *p* < 0.09), score on the National Institutes of Health Stroke Scale (NIHSS) ( *p* = 0.001), lower systolic ( *p* = 0.143) and diastolic ( *p* = 0.21) blood pressures, the Alberta Stroke Program Early CT Score (ASPECTS; *p* = 0.09), vessel occlusion ( *p* = 0.05), use of tenecteplase ( *p* = 0.18), thrombectomy ( *p* = 0.13), and years of experience of the physician ( *p* < 0.001). After multivariate analysis, being treated by a SN (OR: 3.95; 95%CI: 1.44–10.8; *p* = 0.007), NIHSS (OR: 1.07; 95%CI: 1.02–1.12; *p* < 0.002) and lower systolic blood pressure (OR: 0.98; 95%CI: 0.96–0.99; *p* < 0.003) remained significant.

**Conclusion** Treatment by a SN resulted in a higher probability of treating the patient in a DTN time within 20 minutes.

## Resumen

**Antecedentes** La respuesta a la trombólisis intravenosa (TIV) es dependiente del tiempo.

**Objectivo** Comparar los tiempo puerta-aguja (TPAs) de neurólogos vasculares (NVs) contra los de neurólogos no vasculares (NNVs) y médicos emergencistas (MEs), y determinar los elementos asociados a un PTA ≤ 20 minutos.

**Métodos** Análisis observacional prospectivo de pacientes con TIV tratados en Clínica Alemana entre junio de 2016 y septiembre de 2021.

**Resultados** En total, 301 pacientes con TIV fueron tratados. El TPA promedio fue de 43,3 ± 23,6 minutos. Un total de 173 (57,4%) pacientes fueron evaluados por NVs, 122 (40,5%), por NNVs, y 6 (2,1%), por MEs; los TPAs promedios fueron de 40,8 ± 23; 46 ± 24,7 y 58 ± 22,5 minutos, respectivamente. Los TPAs ≤ 20 minutos fueron más frecuentes en pacientes tratados por NVs versus NNVs y MEs: 15%, 4% y 0%, respectivamente ( *odds ratio* [OR]: 4,3; intervalo de confianza del 95% [IC95%]: 1,66–11,5; *p* = 0,004). El análisis univariado demostró que TPA ≤ 20 minutos se asoció con: tratamiento por NVs ( *p* = 0,002), periodo de la pandemia de enfermedad por coronavirus 2019 (COVID-19; *p* = 0,21), tiempo a urgencia ( *p* = 0,21), diabetes ( *p* = 0,142), hipercolesterolemia ( *p* = 0,007), fibrilación auricular ( *p* < 0,09), puntaje en la National Institutes of Health Stroke Scale [NIHSS] ( *p* = 0,001), presión arterial sistólica ( *p* = 0,143) y diastólica menores ( *p* = 0,21), Alberta Stroke Program Early CT Score (ASPECTS ; *p* = 0,09), oclusión de vasos cerebrales (p =0,05), uso de tecneteplase ( *p* = 0,18), trombectomía ( *p* = 0,13) y años de experiencia del médico ( *p* < 0,001). El análisis multivariado demostró que ser tratado por NVs (OR: 3,95; IC95%: 1,44–10,8; *p* = 0,007), el puntaje en la NIHSS (OR: 1,07; IC95%: 1,02–1,12; *p* < 0,002) y la presión arterial sistólica (OR: 0,98; IC95%: 0,96–0,99; *p* < 0,003) se asociaron a TPA ≤ 20 minutos.

**Conclusión** El tratamiento por NVs resultó en un TPA menor y en una mayor probabilidad de tratamiento ≤ 20 minutos.

## INTRODUCTION

Intravenous thrombolysis (IVT) for patients with acute ischemic stroke (AIS) who meet eligibility criteria is efficacious and increases the probability of patient independence at 3 months from the onset of symptoms. 1 2 3 Its effectiveness is time-dependent. The earlier thrombolytic therapy is administered after the onset of symptoms, the lower the risk of hemorrhagic transformation and the better the outcomes at discharge and at 90 days. 1 A door-to-needle (DTN) time, or time elapsed from arrival to the hospital to the administration of the thrombolytic bolus, shorter than 60 minutes is the current recommendation. 4 In highly specialized centers, DTN time can be < 20 minutes. 5 It has been postulated that DTN time based on the results obtained by different specialists dedicated to stroke care should be similar. 6 To our knowledge, there is no study comparing DTN time between stroke neurologists (SNs) and neurologists who provide care in emergency rooms (ERs) but are not dedicated to the management of stroke patients (NSNs).

The aim of this study was to compare DTN time in patients with AIS when treated on arrival by SNs and NSNs and emergency room physicians (EPs). Furthermore, we analyzed which elements were associated with very short DTN time (≤ 20 minutes); this was carried out in a single medical center in Santiago, Chile.

## METHODS

In this prospective cohort study, patients with suspected AIS who consulted the ER of Clínica Alemana of Santiago (CAS) from June 2016 to September 2021 were evaluated by the neurologist on call.

The ER is staffed by six SNs, defined as neurologists with 1 or more years of training in Stroke Units in Chile, Europe, and/or the USA, who regularly take care of stroke patients in the Stroke and/or Intensive Care Units. Additionally, the ER is staffed by five NSNs, who underwent training as neurologist's specialists in areas such as extrapyramidal disorders, and who are knowledgeable of our stroke protocols but who do not treat stroke patients in their practice. In the absence of any of the ER staff neurologists, two SNs or three NSNs who are not ER staff could treat the case.

In patients with AIS, prestroke modified Rankin Scale (mRS) score, sex, age, and cardiovascular risk factors were recorded **.** The time from stroke symptomatology onset (defined as the last time at which the patient was known to be free of any neurological deficit) to arrival to the ER was registered, as was the assessment of stroke severity according to the National Institute of Health Stroke Scale (NIHSS). Systolic and diastolic blood pressures were measured prior to the administration of the thrombolytic bolus.

Patients were then studied with a neuroimaging protocol, which has been previously described 7 and consists of a non-contrast brain computed tomography (NCCT). In patients without contraindications, a spiral computed tomographic angiography of the cervical and intracranial arteries (CTA) and a diffusion-weighted image (DWI) were obtained.

Patients eligible for IVT were treated within a 4.5-hour time window. The alteplase bolus was usually administered after the NCCT and prior to the results of blood parameters, CTA, and DWI. Patients with a wake-up stroke or an unknown time of evolution (UTE) Underwent and additional fluid-attenuated inversion recovery (Flair imaging) and were treated with IVT if the AIS was not visible on this additional imaging or if a CT perfusion (CTP) study demonstrated a good profile. The imaging protocol used for UTE patients was left at the discretion of the attending physician. At our center, there is no established upper NIHSS limit for IVT; patients with low NIHSS may be treated if they had suffered a relevant intracranial large vessel occlusion (LVO), defined as symptomatic disease involving the terminal internal carotid artery or segments M1 or M2 of the middle cerebral artery, the A1 segment of the anterior cerebral artery, the P1 segment of the posterior cerebral artery or the basilar and vertebral arteries, or a neurological deficit that could cause significant disability.

The information obtained from the imaging protocol included NCCT Alberta Stroke Program Early CT Score (ASPECTS), the presence of a hyperdense artery sign and the existence of LVO.

During April of 2020, and due to the coronavirus 2019 (COVID-19) pandemic, our center authorized the use of tenecteplase for AIS treatment in order to evacuate these patients from the ER as soon as possible.

Patients with relevant intracranial large vessel disease are usually treated with bridging therapy with thrombectomy.

Clínica Alemana has two peripheral divisions in Santiago: La Dehesa, located 13.8 kilometers from CAS, and Chicureo, located 24.8 kilometers from the main installation. Both divisions have an ER and could perform our stroke imaging protocol 24/7. They are covered by EPs who frequently work with our stroke team in CAS and who received training in stroke treatment. In the case of an AIS patient, they could discuss the case by telemedicine with the neurologist on call in CAS, and if IVT is provided, the patient is immediately transferred by ambulance to CAS.

For all patients treated with IVT, DTN time was registered according to the type of attending physician (SN, NSN, or EP), staff, or replacement doctors. Finally, it was registered whether the treatment was carried out during regular hours, defined as from 8:00 AM to 7:59 PM or during the night shift, from 8 PM to 7:59 AM, and if it was performed on a weekday or weekend/national holiday.

After the initial treatment, patients were admitted to the Stroke Unit, where additional evaluations could be performed.

Symptomatic intracranial hemorrhage (ICH) was defined as the presence of intracranial hemorrhage on NCCT with an increase of the NIHSS of four or more in the first 36 hours of admission. Favorable outcome was defined as stroke patients with a mRS score ≤ 2 points at the 90-day follow-up. To simulate real-world stroke-treating scenarios, stroke mimics (SM) were not excluded from the analysis.

The Ethics Committee of Clínica Alemana de Santiago/Universidad del Desarrollo approved the registry, and patients, or their relatives, provided written informed consent.

### Data analysis

Door-to-needle times between SNs and non-stroke physicians (NSN and EPs) were compared; the percentage of DTN time > 60 minutes and ≤ 20 minutes for these groups were also calculated.

After excluding those cases treated by EPs (due to the small number of patients), we performed a univariate analysis with Fisher exact test of independence to evaluate the associations of DTN time ≤ 20 minutes with basal mRS, sex, age, cardiovascular risk factors, time to the ER, admission NIHSS, and pretreatment systolic and diastolic blood pressures. Other factors considered were leucoaroisis on NCCT, ASPECT score, the presence of the hyperdense artery sign, bridging endovascular treatment, SN versus NSN, years of experience of the physician, being treated during the day versus the night shift, weekdays or weekends/national holidays, COVID-19 pandemic period versus no pandemic, the use of alteplase versus tenecteplase, and a final diagnosis of SM at discharge were also analyzed.

A logistic regression analysis was performed for those variables that were associated ( *p* < 0.25, Hosmer Lemeshow criteria for fishing variables) in the univariate analysis to select the final multivariate model.

The number of patients who developed an ICH was compared between those treated by SNs, NSNs, and EPs.

Favorable outcome at 90 days was compared between the patients treated by SNs and NSNs.

All analyses were performed with the STATA 1 v 14.0 software (StataCorp LLC, College Stations, TX, USA).

## RESULTS

Between June 2016 and September 2021, 386 consecutive patients with suspected AIS and who were possible candidates for IVT were admitted to our center. Of these, 85 cases did not receive IVT, 31 due to use of an anticoagulant whose effect could not be reversed, 13 because of very mild deficits, 7 suffered from conditions that could increase the risk of ICH, 12 due to non-favorable RAPID (software results) or DWI/FLAIR images, 5 patients because of unfavorable basal medical conditions, and, finally, 17 for a variety of other reasons.

The remaining 301 patients were treated with IVT. Their mean age was 67 ± 18,5 years and 49.2% of these were male. The mean DTN time was 43.3 ± 23.6 minutes, with a median of 37.1 minutes. Only 58 patients (19.2%) had a DTN time > 60 minutes. One hundred seventy-three patients (57.4%) were evaluated by SNs, 122 (40.5%) by NSNs, and 6 (2.1%) by EPs. The baseline characteristics of the patients are shown in Table 1 . Their characteristics were comparable across all groups although a few differences were found: SNs treated fewer patients with diabetes ( *p* = 0.04) and performed more IVT during weekends ( *p* = 0.03) and during the COVID-19 pandemic ( *p* = 0.000). Stroke neurologists had more years of experience ( *p* = 0.001) and used alteplase more frequently ( *p* = 0.000).

**Table 1:** Clinical and radiological variables of patients treated with intravenous thrombolysis in Clínica Alemana

Variables		Stroke neurologist (N = 173)	Non stroke neurologist (N = 122)	Emergency physicians (N = 6)	*P* -value
Mean age, years (SD) Median (IQR)		66.9 (± 19) 69 (53–83)	67.4 (± 17.9) 71(55–82)	62.6 (± 20.1) 70 (47–74)	0.66
Female sex (%)		86 (49.7)	63 (51.6)	4 (66.7)	0.76
Previous	Rankin, mean	0.66	0.68	0.8	0.7
	Median (IQR)	0 (0-1)	0 (0-1)	0 (0–1)	
Hypertension (%)		96 (55.4)	69 (56.5)	2 (33)	0.57
Diabetes mellitus (%)		40 (23.1)	41 (33.6)	0	0.04
Hypercholesterolemia (%)		49 (28.3)	37 (30.3)	1 (16.6)	0.8
Tobacco (%)		30 (17.3)	22 (18)	0	0.73
Heart failure (%)		12 (6.9)	16 (13.1)	0	0.21
Atrial fibrillation (%)		22 (12.7)	15 (12.3)	0	1
Coronary heart disease		19 (10.6)	15(12.3)	0	0.86
Previous antiplatelet		44 (25.4)	30 (24.5)	1 (16.6)	0.96
Time to ER ^†^ , minutes mean ± SD median (IQR)		123 (± 150) 75(45–136)	139 (± 154.5) 85 (48–169)	138(± 139.8) 55 (35–550)	0.3
Admission	NIHSS, mean	8.6	8.2	4.6	0.77
	Median (IQR)	5 (3-14)	6 (3–12)	3 (2-8)	
DTN ‡ (minutes) DTN < 20 min (%)		40.8 ± 23.8 27 (15.6%)	46.3 ± 24.7 5 (4.1%)	58.6 ± 22.8 0	0.004
Systolic BP, mm Hg Diastolic BP, mm Hg mean (SD)		147.1 85.9	150 84.9	141.8 80.8	0.79
NCCT §	ASPECTS, mean	8.7	8.8	9	0.7
	Median (IQR)	10 (10–10)	10 (10–10)	10 (10–10)	
NCCT leukoaraiosis (%)		32 (18.5)	33 (27)	1(16.7)	0.179
Hyperdense arterial, sign (%)		34 (19.6)	29 (23.7)	0	0.37
Arterial occlusion on CTA∥		70 (41.4)	55(45.5)	0	0.07
Complete imaging protocol Performed (NCCT, CTA, DWI¶)		160 (92.4%)	111 (90%)	6 (100%)	0.91
Regular ER staff	Yes	161	111	6	0.000
	No	12	11	0	
ER experience in years (mean) median (IQR)		15.6 ± 2.1 16 (8–30)	10.3 ± 3.8 12(7–21)	8.6 ± 2.8 7 (6–12)	0.001
Treated	Weekdays	119	97	6	0.03
	Weekend/ holiday	54	25	0	
Treated	Daytime	123	78	5	0.358
	Nighttime	50	44	1	
COVID-19 pandemic	Yes	47	27	6	0.000
	No	126	95	0	
Intravenous	Alteplase	128	96	0	0.000
	Tenecteplase	45	26	6	
Thrombectomy		25	26	0	0.19
Stroke Stroke mimic		154 19	111 11	4 2	0.152

Abbreviations: CTA, computed tomographic angiography; COVID-19, coronavirus disease 2019; DWI, diffuse-weighed imaging; IQR, interquartile range; NCCT, mon-contrast computed tomography; SD, standard deviation.

Notes: †Emergency room; ‡door-to-needle; §non-contrast brain computed tomography; ∥spiral computed tomographic angiography of the cervical and intracranial arteries; ¶diffusion weighted imaging.

The mean DTN time was 40.8 ± 23, 46 ± 24.7 and 58 ± 22.5 minutes for SNs, NSNs, and EPs, respectively ( *p* = 0.004). The median DTN time was 35, 39, and 62.5 minutes for these groups. There was no difference between the DTN time prior to and throughout the COVID-19 pandemic period, 43.8 ± 23 versus 41.5 ± 23 minutes.

Door-to-needle time ≤ 20 minutes was more frequently observed in patients treated by SNs when compared to NSNs and EPs: 15%, 4%, and 0%, respectively (odds ratio [OR]: 4.3 (95% confidence interval [95%CI]: 1.66–11.5, *p* = 0.004). Stroke neurologists had more patients with a DTN time ≤ 60 minutes: 82.6%, 79.1%, and 50%, respectively (OR: 4.7; 95%CI: 0.9–24; *p* = 0.125), but these were not statistically significant.

The variables associated with DTN time ≤ 20 minutes are shown in Table 2 , in univariate analysis were: treatment by a SN ( *p* = 0.002), treatment during the COVID-19 pandemic ( *p* = 0.21), time to ER ( *p* = 0.21), presence of diabetes ( *p* = 0.142), hypercholesterolemia ( *p* = 0.007), atrial fibrillation ( *p* = 0.09), NIHSS ( *p* = 0.001), lower systolic ( *p* = 0.143) and diastolic ( *p* = 0.21) blood pressures; ASPECTS on CT ( *p* = 0.09), the presence of a vessel occlusion on CTA ( *p* = 0.05), the use of tenecteplase ( *p* = 0.18), bridging thrombectomy ( *p* = 0.13), and years of experience of the physician who performed the IVT treatment ( *p* = 0.001).

**Table 2:** Univariate analysis correlating door to needle time < 20 minutes with clinical and radiological variables

Variables		DTN ^†^ < 20 minutes N = 32	DTN > 20 minutes N = 263	*P* -value
Mean age, years (SD)		65.4 (± 17.2)	67.1 (± 18.7)	0.61
Female sex (%)		14 (43.7)	135 (51.3)	0.45
Previous	Rankin, mean	0.59 ± 1.1	0.68 ± 1.1	0.63
Hypertension (%)		16 (50)	149 (56.5)	0.57
Diabetes mellitus (%)		5 (15.6)	76 (28.9)	0.142
Hypercholesterolemia (%)		3 (9.38)	83 (31.5)	0.007
Tobacco (%)		4 (12.5)	48 (18.5)	0.63
Heart Failure (%)		3 (6.9)	25 (13.1)	1
Atrial fibrillation (%)		7 (21.8)	30 (11.4)	0.09
Coronary heart disease		4 (12.5)	30(11.4)	0.77
Previous antiplatelet		9 (28.1)	65 (24.7)	0.66
Time to ER, minutes mean ± SD		97 (± 74.9)	133 (± 158.9)	0.21
Admission NIHSS (Mean)		12.7 ± 7.5	7.9 ± 7.3	0.001
Systolic BP, mm Hg Diastolic BP, mm Hg mean (SD)		141.6 ± 28.3 89.2 ± 17.6	149.6 ± 26.3 84.9 ± 17.6	0.143 0.21
NCCT §	ASPECTS, mean	9.3 ± 1.8	9.6 ± 0.8	0.09
NCCT leukoaraiosis (%)		4 (12.5)	61 (23.1)	0.251
Hyperdense arterial, sign (%)		9 (28.1)	54 (20.5)	0.36
Arterial occlusion on CTA		19(59.3)	106 (41)	0.05
Stroke neurologist Neurologist		27 (84.3) 5 (15.7)	146 (55.5) 117 (44.5)	0.002
Staff physician	Yes	29	243	0.72
	No	3	20	
ER experience in years	20 or more	17	38	0.000
	19–15	5	57	
	14.9–10	0	27	
	9.9–5	5	86	
	< 4.9	5	55	
Treated	Weekdays	22	194	0.53
	Weekend/holidays	10	69	
Treated	Day time	24	177	0.428
	Nighttime	8	86	
COVID-19 pandemic	Yes	11 (34.4)	63 (23.9)	0.21
	No	21 (65.6)	200 (76.1)	
Intravenous	Alteplase	21	203	0.18
	Tenecteplase	11	60	
Thrombectomy		9 (28.1)	42 (15.9)	0.13
Stroke Stroke mimic		29 3	236 27	1

Abbreviations: ASPECTS, Alberta Stroke Program Early CT Score; CTA, computed tomographic angiography; COVID-19, coronavirus disease 2019; DWI, diffuse-weighed imaging; ER, emergency room; IQR, interquartile range; NCCT, mon-contrast computed tomography; NIHSS, National Institutes of Health Stroke Scale; SD, standard deviation.

Notes: ^†^ door-to-needle; ‡§ non-contrast brain computed tomography; ∥diffusion-weighted magnetic resonance imaging.

In the multivariate analysis ( Table 3 ), being treated by a SN (OR: 3.95; 95%CI: 1.44–10.8; *p* = 0.007), NIHSS (OR: 1.07; 95%CI: 1.02–1.12; *p* < 0.002), and lower systolic blood pressure on admission (OR: 0.98; 95%CI: 0.96-0.99; *p* < 0.003) remained statistically significant. An addition of 1 point in NIHSS increases by 7% the chance of being treated with a DTN time ≤ 20 minutes; on the other hand, every increase of 10 mmHg of the systolic blood pressure increased the DTN time by 1 minute.

**Table 3:** Variables independently associated with a DTN inferior to 20 minutes. Multivariable analysis.

Variable	OR	95%CI	*p*
Stroke neurologist	3.95	1.44–10.8	0.007
NIHSS	1.07	1.02–1.12	0.002
Systolic blood pressure	0.98	0.96–0.99	0.003

Abbreviations: 95%CI, 95% confidence interval; DTN, door-to-needle; NIHSS, National Institutes of Health Stroke Scale; OR, odds ratio.

An ICH was present in 12 (4.4%) of the 271 stroke patients treated (none of the SM suffered an ICH); 6 of these patients had been treated by a SN (3.8%), 6 (5.4%) by a NSN, and none had been treated by EPs. These differences were not statistically significant ( *p* = 0.63).

Favorable outcome (mRS score ≤ 2) was not statistically different between those treated by a SN: 112 out of 154 patients (72.2%), and NSN 77 (70%) of 110 ( *p* = 0.69). After adjustment for age, presence of diabetes mellitus, NIHSS, systolic blood pressure on admission, and the presence of leukoaraiosis and LVO, the results remained statistically nonsignificant.

On day 90, there was no difference in the distribution of mRS between patients treated by SNs and NSNs ( *p* = 0.69), even for those subjects who had suffered a LVO ( *p* = 0.89).

## DISCUSSION

Intravenous thrombolysis is the only medical reperfusion therapy that improves the outcomes of patients with AIS and its benefits are highly time-dependent.

Guidelines recommend that IVT should be administered with short DTN time, as patients experiencing a large vessel AIS can lose 1.9 million neurons, 14 billion synapses, and 12 km of myelinated fibers every minute. 8

A shorter DNT time not only increased the odds of achieving a modified Rankin score 0 to 1 on day 90, as it also decreased the odds of parenchymal ICH, mortality 9 and even long-term recurrences of stroke. 10 For those patients who could be treated in their first hour of evolution, the odds of satisfactory clinical outcome at 3 months almost doubled. 11

In our experience, were 81%, of the patients were treated within a DTN time < 60, SNs presented a DTN 5.4 minutes shorter when compared to a neurologist from a different subspecialty and 17.4 minutes shorter when compared with EPs. These are important reductions if we consider that a 15-minute decrease in DTN time is associated with a 5% lower odd of in-hospital mortality. 12

Two previous studies, by Lee et al. 6 and by Bates et al., 13 did not show differences between the DTN times for patients treated by a SN or a non-neurologist stroke physician. In contrast with our research, in both studies, the non-neurologist stroke physicians had completed a fellowship in stroke medicine and had considerable experience in this subject. Our NSNs treat AIS patients in the ER on the day they are on call only, but they are not truly stroke experts. Bhatt et al. 14 demonstrated that neuro-hospitalists could administer IVT 23 minutes faster when compared to non-neuro-hospitalists **.** In contrast with our study, the 3 previously mentioned studies had longer median DTN times (between 64 and 104 minutes) and fewer cases. Currently, to the best of our knowledge, there have been no direct comparisons of DTN times between SN and neurologists not dedicated to stroke care, with both being neuro-hospitalists.

In our experience in CAS, EPs were the physicians that showed the longest DTN time. In all cases, telemedicine was activated, and final DTN was 58 ± 22.5 minutes. This result is very similar to that of the TeleStroke Unit (TeleACV) of the Southern Metropolitan Area, Ministry of Health, Chile, in which 115 cases treated with IVT had a median DTN time of 56.5 minutes. 15 As it is not possible to operate this connection inside the scanner room, the EPs have to wait until the all imaging protocol is completed to avoid losing sight of very ill patients, and this extends the DTN time. An experience in Finland demonstrated that well trained EPs could give IVT with remarkable DTN time when the primary responsibility for the treatment was on them, and they gave up on the use of their tele-stroke service. 16

One out of every 10 patients treated in our institution had a DTN time ≤ 20 minutes; this number is low when compared to highly specialized centers, 5 although comparable to those reported in an analysis of 61,426 patients of “get with the guidelines” hospitals, where 5.6% of patients were treated with IVT within 30 minutes of arrival. 17 In our case, the reason for this low number of cases treated with DTN time ≤ 20 minutes is that most of our interventions are focused on the in-hospital management of AIS. Interventions to reduce prehospital delays in IVT delivery, such as educational programs, comprehensive prehospital stroke code, and notification have been shown to be effective in a systematic review and meta-analysis 18 19 but have been difficult to implement as many patients arrive by car or by external independent ambulance systems.

The main factor associated with a DTN time ≤ 20 minute at our institution is being treated by a SN, who is the only physician who is involved in the management of AIS in the ER and the Stroke and Critical Care units, and who are the most experienced and trained in stroke management.

Higher NIHSS scores also correlated with DTN time ≤ 20. This is not surprising as more severe clinical manifestations correlate with more expeditious detection of AIS in the ER. 20 Finally, lower systolic blood pressure at the time of IVT was associated with shorter DTN time. On the other hand, higher blood pressures are not only a contraindication for IVT, but are also associated with the main complication of this therapy, ICH. 21 Additionally, the need for antihypertensive medications before the thrombolysis prolongs DTN times. 22

In our experience, patients treated by a SN show a similar incidence of ICH and functional outcome at 3 months as those treated by NSN. A previous study suggested that when stroke patients are treated by a SN, they have better outcomes. 23 This observation could be a reflection of the small number of cases that we included in our study, but also that, usually, SNs take care of AIS patients as soon as possible, many times when patients are still in the ER and after the initial treatment was done by a NSN.

The presence of a SN in the ER is desirable in view of his expertise in this field. In our experience, this is associated with shorter DTN time; however, SN specialists constitute a limited resource in many countries. Other strategies could also be used in order to reduce the DTN time, such as prenotification of arrival to the Emergency Medical Services, single-call activation of the treatment team, moving the patient directly to NCCT, and administration of alteplase in the scanner. 18

This study has limitations: it represents the experience of a single center and a rather small sample. Additionally, we did not evaluate the reasons why SNs demonstrated shorter DTN time and DTN time ≤ 20 in particular. There are 10% of SM patients, and there was no prehospital stroke notification, which would clearly decrease DTN time. Finally, a stroke fellow is sent to the ER every time a stroke patient arrives, and this could influence the DTN time of patients being treated by a NSN. On the other hand, our research represents a real-world scenario in which the ER is populated by neurologists with different types of training.

In conclusion, SNs demonstrated shorter DTN time and higher probabilities of treating patients in ≤ 20 minutes after arriving when compared to NSNs.
